# Infant Early Gut Colonization by *Lachnospiraceae*: High Frequency of *Ruminococcus gnavus*

**DOI:** 10.3389/fped.2016.00057

**Published:** 2016-06-02

**Authors:** Valeria Sagheddu, Vania Patrone, Francesco Miragoli, Edoardo Puglisi, Lorenzo Morelli

**Affiliations:** ^1^Facoltà di Scienze Agrarie, Alimentari e Ambientali, Istituto di Microbiologia, Università Cattolica del Sacro Cuore, Piacenza, Italy

**Keywords:** *Lachnospiraceae*, babies, *Ruminococcus*, *Blautia*, qPCR

## Abstract

*Lachnospiraceae* is a bacterial family usually isolated from human and mammalian intestinal microbiota. However, its presence and role in the infant microbiota is not fully elucidated. This may be due to the strictly anaerobic behavior of its members that hampers the possibility of culture-dependent enumeration. Here, we report on the presence of this bacterial group, using biomolecular techniques, in stool samples from 25 babies aged between 1 and 24 months. Denaturing gradient gel electrophoresis (DGGE) was used as a first detection step, and data were confirmed by quantitative PCR (qPCR). The DGGE showed the presence of *Lachnospiraceae* in infant fecal specimens and indicated the prevalence of *Ruminococcus gnavus* (*R. gnavus*). The qPCR confirmed the presence of the *Clostridium XVIa* group, *Blautia* genus, and *R. gnavus*, which are the main members of this family. We detected *R. gnavus* in 22 of 25 (88%) samples with a qPCR probe assay. Despite the difficulties associated with their detection and enumeration, *Lachnospiraceae*, and in particular *R. gnavus*, should be included in future studies on the infant microbiota composition.

## Introduction

The bacterial composition of gut microbiota in early life is of paramount relevance for the health of infants and also later on, as colonization by commensal human intestinal bacteria stimulates a range of important functions, from postnatal intestinal development to maintenance of the mucosal barrier and nutrient absorption (1). The massive use of culture-independent, DNA-based techniques has improved our knowledge about the presence of bacterial species not easily cultured. The infant core microbiota is composed of species belonging to the phylum *Firmicutes* (including *Clostridium*, *Enterococcus*, *Lactobacillus*, and *Ruminococcus*) or to the phylum *Bacteroidetes* (including *Bacteroides* and *Prevotella* genera), and these phyla constitute over 90% of the known phylogenetic categories found in the human intestine (2, 3). The use of DNA-based techniques confirmed that breast-fed infants have a microbiota dominated by *Bifidobacterium* (4, 5) with a lower presence of *Escherichia coli* (*E. coli*), *Clostridium difficile* (*C. difficile*), the *Bacteroides fragilis* (*B. fragilis*) group, and lactobacilli than the microbiota of exclusively formula-fed infants (6).

In recent years, members of *Lachnospiraceae*, a family of the order *Clostridiales*, have been detected, using DNA-based approaches, as constituents of mammalian intestinal microbiota, including that of humans (7). *Lachnospiraceae* constitutes one of the major taxonomic groups of the human gut microbiota that degrade complex polysaccharides to short-chain fatty acids, including acetate, butyrate, and propionate, that can be used for energy by the host (8). Other animals commonly harbor *Lachnospiraceae*, with herbivores having a higher abundance than carnivores (9). The wide range of functions carried out by *Lachnospiraceae* may influence their relative abundance in gut communities of different hosts.

The *Lachnospiraceae* family is formed by 24 named genera, including *Ruminococcus*, *Blautia*, *Dorea*, and *Lachnoanaerobaculum* as well as a number of *incertae sedis* strains (10) sharing a high degree of similarity among their 16S rDNA sequences (11, 12). Some members of this family are non-spore-forming, but all of them are strictly anaerobes (12). In human adults, members of this family have been associated with protection against *C. difficile* infections (13) and obesity (14). They are also known as potent short fatty acid producers (15). However, despite their apparent importance, little is known about their presence and possible roles played by these bacteria in the early life of humans.

Members of this family, mainly belonging to the genus *Ruminococcus*, have been identified in the stool samples of neonates and infants (16–18), while it was impossible to detect their presence in 46 babies delivered by natural delivery or cesarean section (19). Their presence was also confirmed in babies fed soy milk (20) or goat milk (21). Results of this latter study suggested a relevant presence of members of the species of *Ruminococcus gnavus* (*R. gnavus*) in babies fed breast milk or goat milk formula compared with babies fed cow milk formula, while *Bifidobacteriaceae* were abundant in the microbiota of infants in all three groups.

The present study aimed to assess the presence of *Lachnospiraceae* as a component of the infant microbiota in a group of 25 infants, focusing on Ruminococci and in particular the presence of *R. gnavus*, from the first month up to the second year of life by means of denaturing gradient gel electrophoresis (DGGE) and quantitative PCR (qPCR). To further assess our hypothesis that *Lachnospiraceae* could be a dominant bacterial group in the infant gut from the first month to the second year of life, qPCR was used to quantitate the total *Bifidobacterium* species for a reference.

## Materials and Methods

### Subjects

Twenty-five infant fecal specimens (age range: 1 month–2 years, mean: 5.6 months, SD: 5.3 months) were prospectively collected to investigate the prevalence of the *Lachnospiraceae* family, particularly of *R. gnavus*. Only one stool specimen was collected for each subject, babies were delivered either vaginally or by cesarean and they were breast-fed or formula-fed, with some in the weaning period. No antibiotic treatment was provided during the 4 weeks before the analyses. In details, fecal samples belonged to 17 vaginal and 8 to C-section delivered babies. Vaginal and C-section delivered babies were divided by feeding mode: respectively, 14 breast-fed and 3 formula-fed for the natural delivered infants, instead, 4 by breast and 4 by formulas for the Cesarean ones. Stool specimens were stored at −20°C until used. The samples collection was a prosecution of a previous study (18). Informed written consent was obtained from the mothers of all subjects, and the study was conducted in conformity with the Helsinki Declaration. The Ethics Committee of the “Ospedali Riuniti” University Hospital, Polytechnic University of Marche, Ancona (Italy) approved the study.

### Fecal DNA Extraction

Stool samples were thawed at room temperature, and bacterial genomic DNA was extracted from 50 mg (wet weight) using the FastDNA™ SPIN Kit for Soil (MP Biomedicals, Switzerland) according to the manufacturer’s instructions (22–24). Extracted DNA was eluted with 100 μl of elution buffer and stored at −20°C until used. Some microbial groups considered to be highly significant in infant intestinal microbiota were investigated with primers previously described (25–28), as reported in Table S1 in Supplementary Material.

### PCR–DGGE

Currently, primers specific for the *Lachnospiraceae* family are not available; therefore, we used those described by Maukonen et al. to amplify the V6 region of the 16S rRNA gene of the *Clostridium coccoides* (*C. coccoides*)–*Eubacterium rectale* (*E. rectale*) group, a bacterial cluster with no taxonomic recognition that includes all genera of *Lachnospiraceae* family (29). The PCR products obtained with the CcocF–GC-CcocR primers (29) were analyzed by DGGE, on 6% (w/v) polyacrylamide gels (37.5/1, acrylamide/bis-acrylamide) with 38–60% linear DNA-denaturing gradients. Electrophoresis was carried out at 120 V, 60°C, for 20 h in an INGENYphor 2 × 2 System (INGENYphor, Goes, Netherlands).

**Figure 1:**
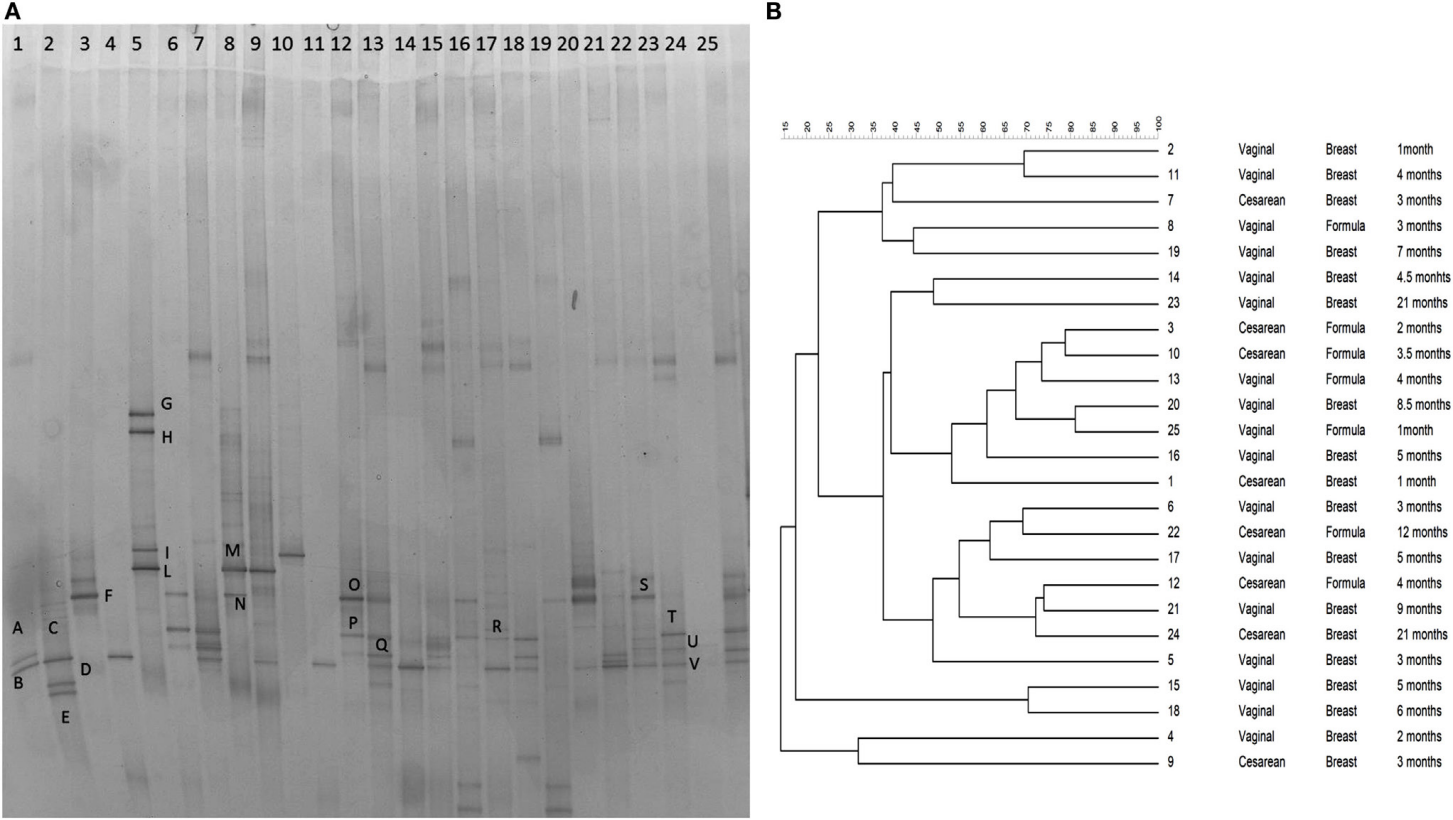
**(A)** PCR–DGGE profiles of 25 samples obtained using the primers CcocF–GC-CcocR. Samples are ordered by age from the youngest (1 month) to the eldest (24 months of age). Bands marked with letters were subjected to sequencing after re-amplification, and the corresponding relative identities were obtained by alignment in GenBank and are listed in Table 1. **(B)** Dendrogram obtained from DGGE patterns by software analysis based on the Pearson’s correlation coefficient with the UPGMA algorithm. Columns indicate the delivery mode, the feeding type, and the age of samples.

The gel was stained with SYBR Green I (Roche, Burgess Hill, UK) (Figure 1A). Subsequently, bands of interest were excised, re-amplified, sequenced (BMR Genomics, Padova, Italy), and then compared with sequences in GenBank (http://www.ncbi.nlm.nih.gov/) using BLAST (30) and the blastn algorithm and the Ribosomal Database Project (31). Fingerprinting II SW software (Bio-Rad Laboratories, Hercules, CA, USA) was used for the analysis of the PCR–DGGE profiles. Dendrograms were obtained by the Pearson’s correlation coefficient, using the Unweighted Pair Group Method with Arithmetic Mean algorithm (UPGMA) (Figure 1B).

### Quantitative PCR

Based on the results of DGGE bands discriminant analysis, qPCR of *Clostridium cluster XIVa*, the *Blautia* genus, and *R. gnavus* was performed. To specifically quantify the *Lachnospiraceae* family, we used (unpublished data) qPCR primers previously described by Newton et al. (32). Unfortunately, the couple of primers and probe employed in the study were targeting the specific phylotype related to *Blautia wexlerae* (*B. wexlerae*). Thus, to prevent the underestimation of the *Lachnospiraceae* family, we quantified the entire *Clostridium cluster XIVa* with the primers reported in Table S1 in Supplementary Material. Furthermore, the total amount of Bifidobacteria was determined by means of qPCR using the StepOnePlus™ Real-Time PCR System (Applied Biosystems Japan, Tokyo, Japan) and the KAPA SYBR^®^ FAST qPCR Kit Master Mix 2× (Biolab Scientifics Instruments SA, Switzerland) or the KAPA probe^®^ FAST qPCR Kit Master Mix 2× (Biolab Scientifics Instruments SA, Switzerland). We quantified the different groups with qPCR reactions under the conditions reported in Table S1 in Supplementary Material. Standard curves were generated from 10-fold dilutions of genomic DNA isolated from collection type strains (Table S1 in Supplementary Material).

## Results

The DGGE profiles showed that the bacterial community of all samples produced different banding patterns; thus, the composition of the *Lachnospiraceae* family was different between subjects. Results suggested that the vertical distribution of the DGGE profiles did not depend on the age of the subject. It was influenced by interpersonal variability. The alignment showed that the most prevalent species recovered in samples were *Blautia luti* (*B. luti*), *Blautia producta* (*B. producta*), *B. wexlerae*, *Lachnoanaerobaculum orale* (*L. orale*), *Dorea formicigenerans* (*D. formicigenerans*), *E. rectale*, and *R. gnavus*, as reported in Table 1. The most interesting result obtained from the DGGE was that *R. gnavus* was present in 16 out of 25 samples (Figure 1A). The 16 positive samples for the presence of *R. gnavus* were from 10 of the 17 babies born by natural delivery and 6 of the 8 babies born by C-section. Furthermore, samples from 11 of the 18 breast-fed babies and 5 of 7 formula-fed babies displayed the band corresponding to *R. gnavus*. Combining the delivery and the feeding conditions: 4 of the 16 positive samples were C-section delivered and breast-fed, 2 were C-section delivered and formula-fed, 7 were vaginally delivered and breast-fed, and 3 were vaginally delivered and formula-fed babies. The DGGE preliminary results suggest that the presence the *R. gnavus* is not strictly dependent on the delivery and feeding mode, and this hypothesis is corroborated by the cluster analysis (Figure 1B).

**Table 1:** **Identification of bacteria belonging to the *Clostridium XIVa* group based on DGGE profiles as shown in Figure 1A**.

Identification	Bands^a^	Accession number	% Similarity
*R. gnavus*	B, C, D, E, V	NR_036800, NR_118690	100
*E. rectale*	N	NR_074634	100
*L. orale*	G, H, I, L, M	NR_118086	99
*B. luti*	A, Q	NR_114315.1	99
*B. producta*	F, O, S, R	NR_113270	99
*B. wexlerae*	T, U	NR_044054	100

*^a^Bands are lettered as indicated on the DGGE gel shown in Figure 1A*.

Quantitative PCR results indicated that the mean number of 16S rRNA gene copies detected in fecal samples using primers for the *Clostridium XIVa* group was 9.507 ± 2.397 (log 16S rRNA gene copies per gram of wet feces), while primers specific for the *Blautia* genus detected 7.863 ± 8.213 log 16S rRNA gene copies per grams of wet feces. *R. gnavus* was present at a level of 8.722 ± 9.289 (log 16S rRNA gene copies per gram of wet feces), and this microorganism was detected in 22 of 25 (88%) samples, apparently due to the higher sensitivity of the PCR technique compared with the DGGE. The quantification of Bifidobacteria by qPCR showed that it is present in all samples at a level of 9.976 ± 10.191 (log 16S rRNA gene copies per gram of wet feces).

## Discussion

*Lachnospiraceae* is a bacterial family known to be abundant in the intestinal ecosystem, and recently, there has been a growing interest in the presence and role of these microorganisms in the adult microbiota. *R. gnavus* was found in the intestinal tract of 90% of adults (2) and sharply increased in pathological conditions, such as inflammatory bowel diseases (IBD) (33). It has been described that the *R. gnavus* ATCC 29149 strain possesses the complete Nan cluster involved in sialic acid metabolism for the production of an intramolecular trans-sialidase (34). Moreover, the analyses of metagenomes confirm that this enzymatic pathway is present in healthy subjects and is predominant in IBD metagenomes (34). It has also been recently demonstrated that *R. gnavus* produces iso-bile acids. The iso-bile acids detoxification pathway influences the growth of one of the predominant genera in the human gut, i.e., the *Bacteroides* (35). These recent studies underline the importance of understanding the biological role of the *Lachnospiraceae* family and, in particular of *R. gnavus*, in the complexity of the human microbiota in babies and adults.

However, the presence and role of *Lachnospiraceae* members, and in particular *R. gnavus*, in the infant gut is still unclear. A deeper understanding of the prevalence and diversity of bacteria in the gut of infants is important for human gut ecology and future nutrition research. In our study, the total number of subjects harboring *R. gnavus*, evaluated using the 16S rRNA gene, were higher compared with other reports in literature (16, 17, 19, 20) (Table S2 in Supplementary Material), supporting the need to develop specific molecular tools for this bacterial group. The difference in subjects positive for the presence of *R. gnavus* could be due to the different DNA extraction methods and different sets of primers used for the DGGE analysis. Several studies (16, 19, 20) found the *Ruminococcus* genus using the DGGE analysis with the universal primers U968-GC-f and L1401-r (36) for the amplification of the V_6_–V_8_ 16S rRNA region. Yu and Morrison (37) reported that this primer set allows a minor recovery of bacterial richness. Other universal primers for the 16S rRNA gene were employed to evaluate the bacterial populations harbored by the infant gut, such as HDA-1-GC and HAD-2 (18). The lower presence of *Ruminococcus* spp. could be attributed to the use of a set of primers targeting the V_2_–V_3_ region of the 16S rRNA of bacteria, not specifically for the *Clostridium XVIa* group like we used. Furthermore, the assessment of different techniques, such as temperature gradient gel electrophoresis, with universal primers (17) could lead to an underestimation of the *Ruminococcus* spp. In the present study, we failed to detect *R. gnavus* 16S rRNA gene sequences in 9 out of 25 stool specimens by DGGE. For only three specimens, we found no presence by DGGE or qPCR, although this could be due to the limit of detection of the molecular techniques or to the coverage of the primers used in this study. This result suggests that *R. gnavus* colonization in the infant intestine is variable across individuals and not strictly linked to the type of feeding or the delivery mode. As reported in a previous study (38), the presence of the *Ruminococcus* genus was identified by a molecular method as another additional anaerobic bacterial group that has to be considered as “dominant” after the very first days of life, together with Bifidobacteria. Our results further support this hypothesis and strongly suggest that the presence of *R. gnavus* is predominant in the infant gut at levels as high as Bifidobacteria and is not dependent on the type of delivery and feeding. This finding is particularly relevant because Ruminococci and Bifidobacteria share metabolic pathways involved in complex sugar degradation (39) and in the degradation of mucin (40). Thus, the presence of high levels of *R. gnavus* is a notable finding and supports the suggestions that the release of sugars by the mucin degradation might be important for succession by other bacteria (41). Further investigations are required to ascertain whether the high level of *R. gnavus* has functional consequences. Blanton et al. suggested a putative role of *R. gnavus* in ameliorating growth and metabolic abnormalities in animals receiving fecal transplantation from malnourished babies aged 6–18 months (42). Their results support the ability of *R. gnavus* in promoting protein synthesis and lean body mass formation instead of amino acid oxidation. The results obtained in a murine model indicate that *R. gnavus* may help preventing malnutrition and clearly support the relevant role of this organism in the assessment of infant gut microbiota. Our pilot study has several limitations that must be acknowledged. Among these, a major limitation is represented by the low number of recruited subjects, and thus, investigations on a wider cohort of babies are necessary to get a deeper insight into the microbial ecology of *R. gnavus* in the gut. Nevertheless, the present study represents the first work supporting using the qPCR technique to quantify the presence of *R. gnavus* in the intestinal tract of infants until the second year of life, and the results support the need for further efforts to verify and elucidate the biological role of this organism in the early stages of life.

## Author Contributions

VS drafted the manuscript, collected samples, and performed DNA extraction and quantitative PCR. VP jointly led the study and revised the manuscript. FM performed the denaturing gradient gel electrophoresis and the cluster analysis and revised the manuscript. EP revised the manuscript. LM conceived and designed the study and revised the manuscript. All the authors read and approved the final manuscript.

## Conflict of Interest Statement

The authors declare that the research was conducted in the absence of any commercial or financial relationships that could be construed as a potential conflict of interest.
