# Supplementary material for: Infant Early Gut Colonization by Lachnospiraceae: High Frequency of Ruminococcus gnavus
Source: Front Pediatr. 2016 Jun 2;4:57. doi: 10.3389/fped.2016.00057 (PMC4889575; doi:10.3389/fped.2016.00057)
Supplement: Supplementary file 1 [file data_sheet_1.docx]

Supplementary Material

**Infant early gut colonization by *Lachnospiraceae*: high frequency of *Ruminococcus gnavus***

**Valeria Sagheddu^1^*, Vania Patrone^1^, Francesco Miragoli^1^, Edoardo Puglisi^1^, Lorenzo Morelli^1^**

*** Correspondence:** Valeria Sagheddu: valeria.sagheddu@unicatt.it

# Supplementary Table

## Supplementary Table 1

Supplementary Table 1: Conditions and thermal protocols for the assessment of the qPCR targeting the 16S rRNA gene.

|  |  |  |  |  |  |
| --- | --- | --- | --- | --- | --- |
| **Group** | **Primer set** | **Primers and probe final concentration** | **Thermal protocol** | **Standard curve** | **References** |
| *Clostridium XIVa* group | ErecF - ErecR | 400 nM | 95°C 10 s | g DNA *E. rectale* DSM 17629 | (25) |
|  |  |  | 60°C 50 s |  |  |
|  |  |  | 35 cycles |  |  |
| *R. gnavus* | Forward primer, Reverse primer, Probe | 200 nM (primers), 100 nM (probe) | 95°C 10 s | g DNA *R. gnavus* ATCC29149 | (26) |
|  |  |  | 60°C 20 s |  |  |
|  |  |  | 35 cycles |  |  |
| *Bifidobacterium* genus | Forward primer, Reverse primer | 300 nM | 95°C 10 s | g DNA *B. infantis* ATCC15697 | (27) |
|  |  |  | 60°C 60 s |  |  |
|  |  |  | 35 cycles |  |  |
| *Blautia* genus | g-Blau-F, g-Blau-R | 200 nM | 95°C 10 s | g DNA *B. producta* | (28) |
|  |  |  | 60°C 50 s | DSM2950 |  |
|  |  |  | 40 cycles |  |  |
|  |  |  |  |  |  |

## Supplementary Table 2

Supplementary Table 2: Recent literature reporting the presence of Ruminococcus spp. in the infant gut.

|  |  |  |  |
| --- | --- | --- | --- |
| **References** | **Total number of subjects** | **Positive subjects for *Ruminococcus spp.*** | **Age period** |
| (16) | 2 | 2 of 2 babies | from birth 10 months |
| (17) | 11 | 1 of 11 babies | before weaning period |
| (17) | 11 | 3 of 11 babies | weaning period |
| (17) | 11 | 6 of 11 babies | post weaning period |
| (18) | 39 | More than half | First month of life |
| (19) | 46 | 0 of 46 babies | term neonates |
| (20) | 12 | 6 of 12 babies | from 3 to 8 months |
| Our study | 25 | 22of 25 babies | From 1 month to 2^nd^ year |
|  |  |  |  |
